# LAPAROSCOPIC DISTAL PANCREATECTOMY WITH OR WITHOUT SPLEEN PRESERVATION: COMPARATIVE ANALYSIS OF SHORT AND LONG-TERM OUTCOMES

**DOI:** 10.1590/0102-672020190001e1461

**Published:** 2019-12-09

**Authors:** Sergio Renato PAIS-COSTA, Guilherme Costa Crispim de SOUSA, Sergio Luiz Melo ARAUJO, Olímpia Alves Teixeira LIMA, Sandro José MARTINS, Orlando J. TORRES

**Affiliations:** 1 Santa Lúcia Hospital, Brasília, DF; Brazil; 2 Brasília Hospital, DF; Brazil; 3 Brasília University, DF; Brazil; 4 Federal University of Maranhão, São Luis. MA, Brazil

**Keywords:** Distal pancreatectomy, Splenectomy, Laparoscopy, Splenic preservation, Surgery, Pancreatic neoplasms, Pancreatectomia distal, Esplenectomia, Laparoscopia, Preservação esplênica, Cirurgia, Neoplasias pancreáticas

## Abstract

**Background:**

Laparoscopic distal pancreatectomy (LDP) is the preferred approach for resection of tumors in the distal pancreas because of its many advantages over the open approach.

**Aim:**

To analyse and compare short and long-term outcomes from LDP performed through two different techniques: with splenectomy vs. spleen preservation and splenic vessel preservation.

**Method:**

Fifty-eight patients were operated and subsequently divided between two groups: Group 1, LDP with splenectomy (LDPS); and Group 2, LDP with spleen preservation and preservation of splenic vessels (LDPSPPSV).

**Results:**

The epidemiological characteristics were statistically similar between the two groups (age, gender, BMI and lesion size). Both the mean of operative time (p=0.04) and the mean of intra-operative blood loss (p=0,03) were higher in Group 1. The mean of resected lymph nodes was also higher in Group 1 (p<0.000). There were no statistic differences between the groups in relation to open conversion, morbidity or early postoperative mortality. The mean hospital stay was similar between groups. Pancreatic fistula (grade B and C) was similar between the groups. The mean of overall follow-up was 37.6 months (5-96). Late complications were similar between the groups.

**Conclusion:**

Both techniques were superimposable; however, LDPS presented, respectively, higher intra-operative bleeding, longer duration of the operation and higher number of lymph nodes resected. No differences were observed in the studied period in relation to the appearance of infections or neoplasm related to splenectomy during follow-up. Maintenance of the spleen avoided periodic immunizations in patients in LDPSPSV. It is indicated in small pancreatic lesions with indolent course.

## RESUMO

**Racional:**

Pancreatectomia distal laparoscópica (PDL) é a abordagem de escolha para ressecção de tumores no pâncreas distal devido a suas muitas vantagens sobre a abordagem laparotômica.

**Objetivo:**

Analisar e comparar os resultados em curto e longo prazo da PDL realizada por meio de duas técnicas diferentes: com esplenectomia vs. preservação esplênica e preservação dos vasos esplênicos.

**Método:**

Cinquenta e oito pacientes foram operados e subsequentemente divididos em dois grupos: grupo 1, PDL com esplenectomia (PDLE) e grupo 2, PDL com preservação esplênica e dos vasos esplênicos (PDLPEVE).

**Resultados:**

As características epidemiológicas foram similares entre os dois grupos (idade, gênero, IMC e tamanho da lesão). Ambas as médias de tempo operatório (p=0,04) e de sangramento intraoperatório (p=0,03) foram maiores no grupo 1. A média de linfonodos ressecados também foi maior no grupo 1 (p<0.000). Não houve diferença em relação a conversão, morbidade ou mortalidade pós-operatória precoce. A média de internação hospitalar foi similar entre os grupos. Fístula pancreática (graus B e C) foi similar entre os grupos. A média de seguimento global foi 37,6 meses (5-96). Complicações tardias foram similares entre os grupos.

**Conclusão:**

Ambas as técnicas foram superponíveis, entretanto a PDLE apresentou respectivamente maiores sangramento intraoperatório, tempo cirúrgico e número de linfonodos ressecados. Não foram observadas diferenças de infecções ou neoplasias relacionadas com esplenectomia durante o período de seguimento. Manutenção do baço evitou imunizações periódicas na PDLPEVE. Ela pode ser indicada em lesões pancreáticas pequenas com curso indolente.

## INTRODUCTION

Laparoscopic distal pancreatectomy (LDP) has rapidly become popular as a preferred treatment method for lesions in the distal portion of the pancreas ^24^ ^,^ ^30^. Since the first laparoscopic pancreatic resection conducted by Cuschieri et al.^4^ in 1994, laparoscopic pancreatic surgery has become highly common worldwide, with diversification of its indications and routine use in medical practice. Although expertise initially only related to resection of small and benign lesions (enucleation), rapid evolution into more complex techniques such as caudal, total and central pancreatectomy and pancreatoduodenectomy has been observed^13^ ^,^ ^14^ ^,^ ^23^. 

Compared with the laparotomic technique, LDP presents several advantages. Among them it gives rise to less postoperative pain, shorter hospitalization, earlier recovery, lower morbidity (both in relation to the abdominal wall and in general) and obvious aesthetic benefits^5^ ^,^ ^6^ ^,^ ^10^ ^,^ ^30^. Thus, despite the technical difficulties inherent to pancreatic surgery, LDP has gradually been included in the therapeutic arsenal in various services around the world, given that its long-term results are similar to those from open surgery, including in cases of malignant disease^12^ ^,^ ^21^. Consequently, various distal pancreatic lesions that are often surgically treated can be treated via laparoscopy. Techniques with or without splenectomy can be conducted, depending on the nature of the lesions (benign or malignant), histological type (neuroendocrine tumors can be removed with preservation of the spleen) and local invasion^1^ ^-^ ^3^ ^,^ ^8^ ^,^ ^16^ ^-^ ^21^ ^,^ ^26^ ^-^ ^29^. 

In turn, laparoscopic distal pancreatectomy with splenectomy (LDPS) is a much more widely used technique because it is easy to perform. Moreover, it is indispensable in cases in which lymphadenectomy of the splenic hilum plays a paramount role both during staging and during specific treatment, such as in treating adenocarcinoma of the pancreas^16^ ^,^ ^30^.

Nonetheless, although tumours of low aggressiveness can be treated via LDPS, spleen preservation would provide a theoretical advantage from the immunological point of view. According to a recent meta-analysis conducted by Nakata et al. ^15^, few studies comparing these different techniques with and without splenectomy and their differences have been conducted, over either the short or the long term. 

Although in a previous study^17^ we showed that laparoscopic distal pancreatectomy with spleen preservation using preservation of the splenic vessels technique (LDPSPPSV) yielded good results, uncertainties relating to the different indications for LDPS and LDPSP remain, regarding the real practical benefit of splenic tissue preservation over the short or long term and other possible advantages not previously studied. Therefore, a comparative study between the LDPS and LDPSPPSV techniques was proposed. 

The objective of the present study was to evaluate, regardless of the indication for each type of the technique (LDPS or LDPSPVP), whether spleen preservation might provide any short or long term difference, and to evaluate whether its preservation might decrease the chances of infection or neoplasia.

## METHODS

This study was approved by the Research Ethics Committees of both institutions where this study was conducted. This was a paired comparative retrospective study between two different LDP techniques: with splenectomy (LDPS) or with spleen and splenic vessel preservation (LDPSPPSV). Sixty-six patients underwent these operations between January 2008 and June 2018. They were respectively divided in two groups: group 1 (LDPS) and group 2 (LDPSPPSV). The inclusion criteria were: patients with radiological (CT or MRI), echo-endoscopic and histological diagnosis of pancreatic neoplasms (cystic, solids, or both); absence of distant metastasis (hematogenic or peritoneal); only patients which underwent a potentially curative resection; and adults. The exclusion criteria were: patients with loss of their follow-up, and the ones submitted to Warshaw´s technique. This way, a total of 58 patients were selected since that eight were excluded (six underwent Warshaw’s technique and another two were loss in follow-up). These 58 patients presented the following distribution: group 1 (n=32), and group 2 (n=26). The decision in performing splenectomy was oncologic or tactical principles. Was oncologic in those patients in which presented adenocarcinoma or neuroendocrine (moderated-high grade) at histological evaluation or still there was tumoral comprising either the spleen or the splenic vessels. In other hand, it was tactical when during intraoperative evaluation there were many strong tumor adherences within spleen or splenic vessels leading a difficulty in progressing the preservation of these structures, besides that splenectomy was also performed when the lesion was positioned in a very distal localization close splenic hylum and additionally was very large.

 A specific protocol was drawn up for following up cases of distal pancreatic lesions in which distal pancreatectomy either with and without splenectomy was performed, with the objective of evaluating the results over the short term (duration of the operation, bleeding, transfusion, weight of the surgical specie, number of lymph nodes resected, conversion rate, morbidity and mortality) and over the long term (recurrence, survival and any late infectious or no complications). 

### Preoperative preparation 

All patients underwent preoperative evaluation by means of computed tomography and magnetic resonance imaging in order to estimate lesion size and whether there was any invasion of major vascular structures or other viscera that would contraindicate the laparoscopic procedure. Some cases underwent endoscopic ultrasound with biopsy to define the type of lesion and surgical indication. Neuroendocrine tumors were staged by using octreotide scintigraphy (octreo-scan) or more recently PET-CT with gallium-68 (DOTA-TATE), when available. Among patients for whom splenectomy was initially considered for oncological or even tactical reasons, all of them were vaccinated during their preoperative period against encapsulated bacteria (*Pneumococci*, *Haemophilus* and *Meningococci*), and human influenza viruses. 

### Surgical technique

**FIGURE 1:**
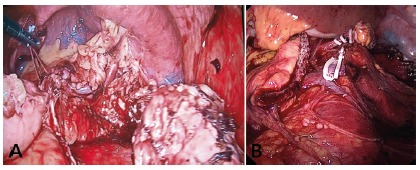
Final aspect of the procedure: A) LDPSPPSV (stump of pancreas was stapled and splenic vessels were preserved); B) LDPS (stump of pancreas was stapled and splenic vessels were ligated with Hemolok clips)

**FIGURE 2:**
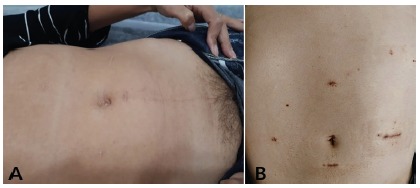
Late results: A) LDPS with position of the portals and incision of Pfannenstiel; B) LDPSPPSV with position of the portals and enlargement of the portal of 12 mm in the right flank for removal of the surgical specimen

**FIGURE 3:**
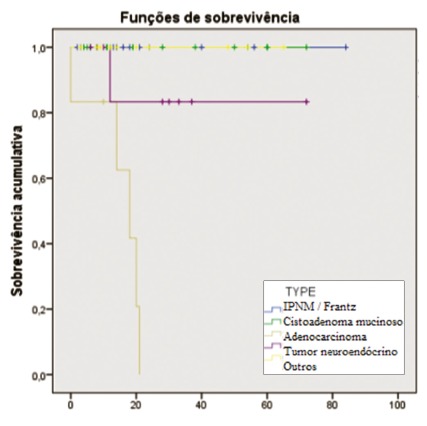
Survival in relation to the type of lesion operated

The surgical technique used in the present study was standardized by the team in all procedures and was described in a previous publication^17^. Essentially, four or five portals were used with 30^0^ optics in a central position (Figures 1A and 1B).The final aspect of the surgeries can be observed in Figures 2A and 2B). In pancreatic stumps, in all cases, a thin tubular drain to the exterior was positioned in the left flank through the 5 mm portal. The surgical specimen was removed in an Endobag or glove more usually through a small low transverse incision of Pfannenstiel type (n=30) as shown in Figure 3A or more rarely a left subcostal incision (n=2) in patients which underwent splenectomy. To the patients which underwent spleen preservation, the surgical specimen was always removed through a widening of the 12 mm portal on the right flank (Figure 3B).

### Postoperative care

During the immediate postoperative period, all the patients were sent to intensive care units. The output from the tubular drain was measured every day and was sent to a laboratory for assaying of amylase on the third day. If the amylase concentration found was more than three times the serum value, the patients were diagnosed as presenting a pancreatic fistula. Once this diagnosis was confirmed, the fistula was classified as type A, B or C, as defined by the International Study Group for Pancreatic Fistula. Individual treatment was then provided for each case, in accordance with the clinical and imaging findings, and the availability of methods within each service. Specifically in cases in which splenectomy was performed incidentally during surgery, all patients were vaccinated against the above mentioned germs after surgery. 

### Postoperative follow-up

All patients were followed up every three months during the first two years and subsequently every six months until the fifth year, when it became annual thereafter. During each return visit, in addition to a thorough clinical examination, full laboratory tests were conducted, including fasting glycemia and glycemic curve, case-pertinent tumor tracers (CA 19-9, CEA, CA 125 and chromogranin A), abdominal tomography with contrast or nuclear magnetic resonance imaging with contrast, and scintigraphy traced with somatostatin analogues (octreoscan) for neuroendocrine pancreatic tumor. All patients were alerted regarding the risk of infection and neoplasia subsequent to surgery, and they were all asked to contact the principal investigator of this study via telephone or e-mail, if these complications were observed during the follow-up period. Specifically, for cases of malignant neoplasm, for survival evaluation purposes, the period considered was between the date of the operation and the date of death due to cancer or the date of the last return to the doctor’s consultation office. 

### Statistical analysis 

The variables of interest were described using frequency tables (categorical and ordinal data), medians and intervals (asymmetrical continuous data) or means and standard deviations (asymmetrical continuous or normally distributed data). For comparison purposes between groups, the ANOVA test was used for continuous parametric variables and Fisher’s exact test was used for nominal non-parametric variables. Survival analysis was calculated using the Kaplan-Meier method, and univariate and multivariate analyses were conducted to evaluate factors that were independent of worse final prognosis. Survival functions were compared between subgroups using the log-rank test (for two groups) or the generalized Wilcoxon test (for three or more groups). In the multivariate analysis, total survival time was obtained using the Cox proportional-hazards model. The effect of the variables was also estimated using the proportional-hazards model (hazard ratio). p<0.05 was considered statistically significant. The SPSS 18 software for Windows (PASW) was used for calculations. 

## RESULTS

The epidemiological variables are described in Table 1. No statistically significant differences in these variables were observed between the groups except by duration of operation, intra-operative bleeding, and number of resected lymph nodes (Table 1).

**TABLE 1:** Epidemiological characteristics and early results

Groups	1 (Splenectomy)	2 (Spleen preservation)	p
n	32	26	0.87
Age*	51.0 years (20 - 78)	47.9 years (21 - 75)	0.43
Gender			
Female	20 (62.5 %)	17 (65.8 %)	0.85
Male	12 (37.5%)	12 (34.6 %)	
Comorbidity	8 (25 %)	6 (23 %)	0.77
ASA II	8 (25 %)	6 (23 %)	0.77
BMI*	28.5 kg/m² (18.3 - 38.3)	25.6 kg/m² (18 - 38.8)	0.06
Lesion size*	4.9 cm (2 - 12)	4.3 cm (1.8 - 7.5)	0.2
Duration of operation*	179.9 minutes (70 - 360)	144.1 minutes (90 - 200)	0.04*
Bleeding*	244.11 ml (0 - 1000)	119.2 ml (50 - 600)	0.03*
Resected lymph nodes*	7.07 (3-12)	2.72 (1-6)	0.000*
Weight of surgical specimen**	162.3 gr (85.1-565.3)	161.5 gr (81,3-358.5)	0.76
Duration of hospitalization*	5.4 days (2 - 13)	4.8 days (2 - 14)	0.43
Conversion	2 (6.2 %)	1 (3.8% )	0.59
Postoperative complications	7 ( 22 %)	6 (23 %)	0,93
Mortality	1 (3.4%)	0	0.31
Positive margins	2 (6.8%)	1 (3,8%)	0.66
Pancreatic fistula (grades B and C)	4 (12.5%)	3 (10.3%)	0.76

*=Variables described in means, *”*=spleen excluded

The etiology of the treated pancreatic lesions is shown in Table 2. 

**TABLE 2:** Etiology of pancreatic lesions

Histological time	Group 1	Group 2
Adenocarcinoma	5 (17.2%)	0
Mucinous cystadenocarcinoma	1 (3.4%)	0
Mucinous cystadenoma	9 (31%)	7 (24.1%)
Serous cystadenoma	3 (10.3%)	7 (24.1%)
IPMN	3 (10.3%)	7 (24.1%)
Neuroendocrine tumor	6 (20.6%)	5 (17.2%)
PSCT (Frantz)	2 (6.8%)	2 (6.8%)
Accessory spleen	0	1 (3.4%)

IPMN=intraductal papillary mucinous neoplasia; PSCT=pseudopapillary solid cystic tumor

Blood transfusion was only necessary in one patient in group 1 (multivisceral resection with conversion due to mucinous cystadenocarcinoma). All of the open conversions were due technical difficulty to continue by means of laparoscopic approach being that in two of these cases, both patients underwent previous gastroplasty for obesity treatment. The only death was in a case of a 12 cm mucinous cystadenocarcinoma that encompassed the transverse colon and spleen, for which multivisceral conversion and resection were necessary. The patient died on the third postoperative day due to cardiovascular complications. Global overall morbidity of this series was 22% (n=13), regarding postoperative complications there was no statistically significant difference between groups (n=0,93). These complications underwent the following distribution by Clavien-Dindo’s Classification: grade I (n=6), grade II (n=3), grade III or more (n=5). Four cases of pancreatic fistulas were observed in group 1 and three cases in group 2 during the postoperative period and no statistically significant difference was observed in relation to this variable (p=0.76). The treatments for these fistulas were as follows: maintenance of the drain in the fistulous passage (n=3); image-guided puncture of the accumulation along the fistulous route with exteriorization of a “pig-tail” drain (n=2); laparotomy for draining intra-abdominal accumulation, evolving with persistent debit, which was resolved after endoscopic pancreatic papillotomy with passage of a prosthesis (n=1); and pseudocyst puncture (late fistula) by means of echoendoscopy and passage of a prosthesis (n=1). In all of these patients except the last one, octreotide and parenteral/enteral nutrition were administered until the fistulas had closed, which occurred after 7 to 38 days (median 18). Histopathological analysis showed that positive margins were present in two cases (6.8%) in group 1 (both cases consisted of adenocarcinoma) and in one in group 2 (neuroendocrine tumor); however, there was no statistically significant difference between groups (p=0.66). 

Among the late complications, there were five complications in each group and no statistically significant difference was observed regarding the number of complications between the groups (Table 2). The complications found comprised segmental splenic infarction without clinical repercussion that was diagnosed through imaging (n=1); glucose intolerance (n=4); diabetes mellitus type 2 (n=2); and exocrine insufficiency (n=3). There were no late infectious complications in any patient, or any post-splenectomy sepsis or appearance of neoplasia during the follow-up period. All patients who underwent splenectomy were periodically vaccinated against encapsulated germs every five years, and against influenza annually. 

**TABLE 3:** Late results

Groups	1 (Splenectomy)	2 (Spleen preservation)	p
n	32	26	
Late complications	5 (15.6%)	5 (19.2%)	0.93
Follow-up	43.5 months (5 - 96)	31.7 months (12 - 72)	0.35
Recurrence	6 (20.68%)	0	0.01*

The mean follow-up period was 43.5 months (5-96) in group 1 and 31.7 months (12-72) in group 2. Recurrence was observed in six patients in group 1, among whom five had adenocarcinoma that later evolved to death, and one patient had a neuroendocrine tumor. The latter presented liver metastases and at his late return he was using octreotide; he had stable disease. The recurrences among the patients with adenocarcinoma presented the following distribution: peritoneum (n=2), liver (n=2) and multiple (n=1). No patient in group 2 had recurrence of the disease. The overall survival curve estimated using the Kaplan-Meier method in relation to histological type can be observed in Figure 3. Group 1 presented lower overall survival and greater long-term recurrence, and these differences were statistically significant. However, the results from univariate and multivariate analyses showed that this finding was clearly associated with the adenocarcinoma histological type, which was, alone, an independent prognostic factor for mortality over the long term (hazard ratio 41.7; p=0.001).

## DISCUSSION

The safety and effectiveness of laparoscopic distal pancreatectomy for treating pancreatic neoplasia has now been proven. This procedure is an excellent choice, compared with open surgery, regarding oncological radicality and long-term results^28^ Additionally to the clear advantages relating to lower morbidity caused by incisions, this access route presents less pain during the postoperative period, shorter hospitalization and earlier recovery to resume work and daily activities^5^ ^,^ ^6^ ^,^ ^10^ ^,^ ^17^ ^,^ ^24^ ^,^ ^30^.

Several studies on LDP have been conducted and, although this technique has become a feasible option, especially over the past decade, there are still a couple of issues that remain current: spleen preservation, either with or without ligature of splenic vessels and control over the fistula of the remnant pancreas^5^ ^,^ ^10^. The main theoretical advantage regarding laparoscopic distal pancreatectomy with spleen preservation (LDPSP) is that this would maintain the immunological functioning of the splenic tissue and hence would diminish infectious and neoplastic conditions. Absence of this organ has been widely correlated with presence of infections caused by bacterial germs, mostly by those that are encapsulated, such as *Meningococci, Pneumococci* and *Haemophilus*. Its absence has also been correlated with rare but extremely lethal cases of post-splenectomy sepsis. In addition, the spleen has an important hematopoietic function, relating both to production of red blood cells and lymphocytes, besides that the destruction of senescent red blood cells. Lastly, maintenance of this organ leads to an economic advantage, through reducing the costs of treating infections and expenditure relating to periodic repeated immunization against certain infectious agents. This also diminishes the natural emotional distress among patients, regarding both repeated immunization and the chances of getting opportunistic infections. In addition, splenectomy has also been correlated with thrombocytosis and thrombosis of the splenic and portal veins. Therefore, preservation of the spleen also reduces the chance of occurrences of these specific complications that relate to the act of the splenectomy itself ^17^ ^,^ ^24^ ^,^ ^29^. 

Although there are certain limitations to the indications for using laparoscopic distal pancreatectomy techniques involving spleen preservation (such cases need to be benign or present low degrees of aggressiveness, for example), use of such techniques has recently become more widespread due these multiple advantages. Besides that the preservation of this organ also directly contributes towards improving patients’ quality of life, since this avoids the need for frequent immunization against these germs. Moreover, should be also considered the economic advantage because it reduces the need for additional expenditure on vaccines and, theoretically, the need for hospitalizations relating to infections caused by the abovementioned germs, along with mortality relating to post-splenectomy sepsis^5^ ^,^ ^28^ ^,^ ^29^. At present study we could observe these advantages in regarding the preservation of the spleen in group 2.

Although these techniques are theoretically more complex and demand greater experience and knowledge from surgeons, spleen preservation should be considered as the first choice whenever possible^3^ ^,^ ^5^ ^,^ ^21^. It can be achieved through two different surgical techniques: 1) with splenic vessel preservation (Kimura’s technique)^11^; or 2) without splenic vessel preservation (Warshaw’s technique)^25^. In the latter, splenic vessels are ligated and splenic irrigation is only maintained through short gastric vessels. In general, whenever possible, our team chooses not to undertake spleen preservation through Warshaw’s technique. Although this technique is considered to be simpler and faster, in addition to requiring less expertise and involving less intra-operative bleeding, it has been associated with larger numbers of specific post-operative complications relating to splenic vessel ligature, such as splenic infarction and gastric fundus varices due to left portal hypertension^17^ ^,^ ^28^ ^,^ ^29^. 

LDPSPPSV or Kimura’s technique has been little studied. Only small case series have been published and, according to a recent meta-analysis by Yongfei et al. ^28^, there has been geographical variation regarding the choice of this type of technique. In the West, most studies on LDP have involved splenectomy or spleen preservation using Warshaw’s technique. However, in the East, a preference for Kimura’s technique can be observed. In the present authors’ opinion, these two techniques overlap and surgeons need to be familiar with both of them, so that they are able to choose to use one rather than the other, according to the specific type of case. 

Recently, in a previous study, we achieved good results using preservation of splenic vessels’s technique in selected cases, such as small benign or low-grade lesions that occurred in patients with favorable anatomy and physical conformations^17^. However, one issue that motivated us to conduct the present study was to evaluate whether, from a practical point of view, spleen preservation or non-preservation might make any important difference in relation to either the early or the late postoperative period, and whether this might have any implication regarding these patients’ immunity. 

A few studies comparing techniques with and without spleen preservation via laparoscopy have been published and several issues have been raised regarding differences that might exist, especially in relation to the potential benefits from spleen preservation. In a recent meta-analysis, Nakata et al.^15^ observed the following differences when the spleen was preserved: less bleeding, shorter duration of the operation, fewer pancreatic fistulas and fewer infectious complications, regardless of the technique used (Warshaw or Kimura), when compared with LDPS. 

In Brazil, at our knowledge, this is the first comparative study between the two laparoscopic distal pancreatectomy techniques with and without spleen preservation, according to the splenic vessel preservation technique. As Farah et al.^9^ as Machado et al.^13^ described a case series of laparoscopic distal pancreatic procedures with spleen preservation; however, they did not specify the technique or the specific results from this group. In the present study, similarities between the two methods were observed. However, as also observed by Nakata et al.^15^, presented a shorter surgical duration and less bleeding compared with the group that underwent splenectomy (LDPS). The number of lymph nodes resected was also statistically lower (p<0.000), perhaps due to a limitation of the technique itself, given that lymphadenectomy of the splenic hilum is difficult mainly without splenectomy. Contrary to what was found by Nakata et al.^15^, who observed more pancreatic fistulas in the group that underwent splenectomy, the prevalence of this complication in the present study was similar between the groups.

Also contrary to what was found by Nakata et al.^15^, the number of infectious complications in the present study was not larger, nor did neoplasms appear in the group that underwent splenectomy (LDPS group 1) during the follow-up period. However, this finding should be interpreted with caution because it can perhaps be explained by the small number of patients in the sample, the shortness of mean follow-up period or even a diagnostic failure during the follow-up period. The rates of occurrence of other long-term pancreas-specific complications, such as endocrinal and exocrinal insufficiencies, were similar between the groups. This may be attributed to the quantity of pancreas tissue resected (i.e. the weight of the fresh specimens), which was similar between the groups (p=0.76). Although there was higher long-term mortality in group 1 (with splenectomy), it was directly related to the frequency of adenocarcinoma etiology in this group. This was a confounding variable in this regard, and it was a prognostic factor that did not depend on mortality after its inclusion in the multivariate analysis on this sample. This finding was already expected, because LDPSPPSV was not performed on patients with this histological pattern, given that this histological type has been considered to be a contraindication for spleen preservation. Splenectomy in cases of adenocarcinoma in the body and caudal regions has also been described as a standard procedure for treating this histological variant in according with the literature^16^.

From a practical point of view, although not many differences were observed between the two techniques, over either the short or the long term, performing LDP with spleen preservation using the splenic vessel preservation technique (LDPSPPSV) whenever pertinent and possible remains preferable, in our view. Its advantages outweigh its risks, therefore we should elect this technique because it has favoured both less bleeding and operative time, besides that has avoided specific complications that are associated with the act of splenectomy itself (like thrombocytosis or even thrombosis of the splenic or portal vein) besides that immunological advantage. This approach avoids frequent immunization consequent to absence of the spleen, thereby saving financial resources, and it improves these patients’ emotional state in relation to the anxiety caused by concerns about these periodic vaccinations and the higher chance of acquiring infections and neoplasias. LDPSPPSV’s technique also seems to present clear advantages over Warshaw’s technique, such as reduction of the chances of future splenectomy due to splenic infarction and gastric fundus varices due to segmental portal hypertension. In summary, LDPSPPSV is worth be attempted, mainly in small pancreatic lesions with indolent course. 

## CONCLUSION

The two techniques overlapped, except for higher intra-operative bleeding, longer duration of the operation and higher number of lymph nodes resected in LDPS. Over the long term, no differences were observed between the groups regarding complications, such as exocrinal-endocrinal insufficiency, infections or appearance of malignant neoplasia. Preservation of the spleen avoided any need for periodic immunization in LDPSPPSV and it is worth to be attempted, mainly in small pancreatic lesions with indolent course. 
